# Multifunctional bimetallic MOF with oxygen vacancy synthesized by microplasma for rapid total antioxidant capacity assessment in agricultural products

**DOI:** 10.1016/j.fochx.2024.101247

**Published:** 2024-02-20

**Authors:** Yi Xia, Juan He, Long Tang, Miao Hu, Jie Zhou, Yao-Yu Xiao, Zhi-Chao Jiang, Xue Jiang

**Affiliations:** a Key Laboratory of Land Resources Evaluation and Monitoring in Southwest, Ministry of Education, College of Chemistry and Materials Science, Sichuan Normal University, Chengdu 610068, China; b School of Mechanical Engineering, Sichuan University, Chengdu 610065, China; c State Key Laboratory of Polymer Materials Engineering, Sichuan University, Chengdu 610065, China

**Keywords:** Bimetallic MOF, Microplasma, Multiple enzyme activities, Total antioxidant capacity

## Abstract

The assessment of total antioxidant capacity (TAC) is crucial for evaluating overall antioxidant potential, predicting the risk of chronic diseases, guiding dietary and nutritional interventions, and studying the effectiveness of antioxidants. However, achieving rapid TAC assessment with high sensitivity and stability remains a challenge. In this study, Ce/Fe-MOF with abundant oxygen vacancies was synthesized using microplasma for TAC determination. The microplasma synthesis method was rapid (30 min) and cost-effective. The presence of oxygen vacancies and the collaboration between iron and cerium in Ce/Fe-MOF not only enhanced the catalyst's efficiency but also conferred multiple enzyme-like properties: peroxidase-like, oxidase-like, and superoxide dismutase mimetic activities. Consequently, a simple colorimetric assay was established for TAC determination in vegetables and fruits, featuring a short analysis time of 15 min, a good linear range of 5–60 μM, a low detection limit of 1.3 μM and a good recovery of 91 %-107 %. This method holds promise for rapid TAC assessment in agricultural products.

## Highlights

• A multifunctional bimetallic MOF was synthesized by microplasma.
• The Ce/Fe-MOF with oxygen vacancies demonstrated multiple enzyme-like properties.
• The Ce/Fe-MOF offered a rapid and cost-effective approach to TAC determination.
• It is a promising tool for rapid TAC assessment in agricultural products.

## 1 Introduction

Vegetables and fruits are important components of our daily diet, containing a significant amount of antioxidants such as vitamin C, vitamin E, carotenoids, and polyphenols (Lako et al., 2007). These substances possess strong antioxidant abilities, capable of scavenging free radicals, reducing oxidative stress, and protecting cells from oxidative damage. They also have various health benefits, including anti-tumor, blood pressure-lowering, blood glucose-lowering, and lipid-lowering effects (Jideani et al., 2021). Total antioxidant capacity (TAC) represents the overall antioxidant capacity of all antioxidant substances in food and serves as an important quality indicator for vegetables and fruits. The types and concentrations of antioxidants present in vegetables and fruits exhibit variations based on factors like variety, cultivation conditions, harvest methods, storage practices, and processing techniques. Consequently, the implementation of portable, highly sensitive, and expeditious Total Antioxidant Capacity (TAC) testing proves advantageous. Such testing not only yields valuable insights into the nutritional composition but also facilitates the monitoring of antioxidant capacity reduction throughout production and processing. Additionally, it enables tracking nutrient loss during food storage, serving as a means to contribute to ensuring consumer health and safety. TAC is generally evaluated by the ability of antioxidants in the sample to reduce a standard oxidizing agent. The typical methods for detecting TAC include ABTS (2,2′-azino-bis(3-ethylbenzothiazoline-6-sulfonic acid)) assay (Zhang et al., 2022), FRAP (ferric ion reducing antioxidant power) assay (Hanuka-Katz et al., 2022). These methods are often combined with other techniques such as high-performance liquid chromatography (HPLC) (Zhang et al., 2015), and electrochemical analysis (Tortolini et al., 2018). Nonetheless, these techniques inherently suffer from some drawbacks, including large instrument size, poor portability, unfavorable real-time field analysis, and extended detection time (more than hours) (Hoyos-Arbeláez et al., 2017). Furthermore, antioxidants are prone to oxidation and damage in ambient air, presenting a significant challenge for swift Total Antioxidant Capacity (TAC) assessment. Consequently, there is a critical need to explore alternative methods that offer enhanced efficiency and practicality, thereby advancing the capabilities of TAC detection.

TAC colorimetric detection had earned considerable attention owing to their simplicity, affordability, rapid analysis speed, relatively stable results, and versatility across various sample types. Notably, sensing methods utilizing Metal-Organic Frameworks (MOFs) present several advantages attributable to the expansive surface area, tunable physicochemical properties, porous structure, and diverse compositions inherent in MOFs. (Wang et al., 2019, Wang and Astruc, 2019). However, MOFs usually face challenges such as low catalytic activity, single active site, and poor stability in the field of catalysis. Toward this, various methods have been proposed to improve the catalytic activity of MOFs such as introduction of oxygen vacancies, structural modification, optimization of synthesis methods, and metal coordination regulation (Chen et al., 2020, Chen et al., 2020, Wang et al., 2020, Wang et al., 2020, Wu et al., 2022, Zhang et al., 2019). Moreover, several studies have found that bimetallic MOFs generally exhibit better sensing performance than single-metal MOFs due to the synergistic effects, electronic effects, and the abundance of unsaturated metal sites (Masoomi et al., 2019). The introduction of metal nodes into Metal-Organic Framework (MOF) materials can generate numerous oxygen vacancies, acting as reactive sites that enhance catalytic activity. Moreover, the incorporation of external metal nodes into MOF materials can create low-coordinated structures, exposing metal active sites and thereby improving catalytic performance (Hu et al., 2023). Hence, the synthesis of bimetallic organic framework nanozymes is highly desirable for TAC detection.

The synthesis method of bimetallic organic framework MOFs greatly affects their structure and catalytic performance. In recent years, there has been significant attention on the synthesis of nanomaterials using microplasma techniques (He et al., 2022), because microplasma exhibit unique properties such as high electric field strength, high reactivity, high energy density, low energy consumption, and controllable operation (Tachibana, 2006). It facilitates diverse unconventional chemical reactions, including electrochemical reactions, redox reactions, dissociation reactions, and ion exchange reactions. These reactions are often challenging to achieve under ambient conditions but can be efficiently conducted in microplasma environments. In the preliminary work of our research group, it was found that microplasma can be used not only to synthesize MOFs, COFs and other inorganic nanomaterials (He et al., 2021, Jiang et al., 2019), but also for enhancing the catalytic performance of MOFs (Xia et al., 2022). Although we have reported that microplasma can enhance the catalytic activity of single-metal Ce-MOF, there is an urgent need to explore higher-activity bimetallic MOF materials for the determination of TAC with high sensitivity and stability.

In this study, the bimetallic Ce/Fe-MOF was one-step synthesized using microplasma for high-sensitivity detection of TAC. Compared to single-metal Ce-MOF and Fe-MOF, Ce/Fe-MOF demonstrated superior catalytic performance. Due to its two redox couples and abundant oxygen vacancies, Ce/Fe-MOF exhibited multiple enzyme-like activities, including peroxidase-like, oxidase-like, and superoxide dismutase mimetic activities, offering enhanced prospects for TAC determination. We established a simple colorimetric assay based on Ce/Fe-MOF using 3,3′,5,5′-tetramethylbenzidine (TMB) as a substrate. When TMB was catalyzed by Ce/Fe-MOF, it turned blue (oxTMB), and upon addition of ascorbic acid (AA), glutathione (GSH), and l-Cysteine (Cys), it became colorless (TMB). This assay provided several advantages, such as short analysis time (15 min), high sensitivity, excellent selectivity, and ease of operation. This work developed a facile method for measuring total antioxidant capacity (TAC), which was successfully applied to fruits and vegetables.

## 2 Material and methods

### 2.1 Materials and reagents

Pericerium sulfate tetrahydrate, terephthalic acid (H_2_BDC), 30 % hydrogen peroxide (H_2_O_2_), 3, 3′, 5, 5′-tetramethylbenzidine (TMB), N, *N*-dimethylformamide (DMF), P-benzoquinone (BQ), Isopropanol (IPA), l-Histidine (HD), Ethylenediaminetetraacetic acid (EDTA), 1,2,3-Benzenetriol, Absolute ethanol, Potassium chloride, Sodium chloride, Calcium chloride, Glucose (Glu), α-Fructose (Fru), α-Lactose (Fru), Glycine (Gly), Glutamic acid (Glu), l-Histidine (His), l-Tryptophan (L-Tr), Glutamine (l-Glu), Tyrosine (L-Ty), Ascorbic acid (AA), Glutathione (GSH), l-Cysteine (Cys), fluorescein (FL), 2,20-Azobis (2-amidino propane)dihydrochloride (AAPH), Trolox (2,5,7,8-tetramethylchroman-2-carboxylic acid) and Potassium bromide were purchased from Cologne Chemical Reagent (Chengdu) without further treatment. 5,5-Dimethyl-1-pyrrolidine *N*-oxide (DMPO) was purchased from Aladdin Biochemical Technology Co., Ltd. (Shanghai). Ag/AgCl electrodes and Pt sheets were purchased from Shanghai Yuechi Electronic Technology Co., Ltd. (Shanghai). NaAc-HAc buffer (0.1 M, pH 4) was purchased from Beijing North Weiye Metrology Technology Research Institute Co., Ltd. (Beijing). Superoxide dismutase (SOD) activity assay kit (Sangon Biotech, Shanghai, China). TAC assay kits using the ABTS and FRAP method were procured from Sangon Biotecch (Shanghai, China). The ultrapure water used to prepare the solution comes from the water purification system (Chengdu Ultrapure Technology Co., Ltd.).

### 2.2 Instrumentation

The crystal structure of the material was determined using a RigakuD/MAX2550 x-ray diffractometer (XRD) with scanning speeds of 40 kV, 30 mA, and 6° min^−1^. The morphology of the material was measured by a 250 quanta field emission scanning electron microscope (FESEM). The microstructure and elemental composition were investigated using a JEM-2100 TEM (Jeol Ltd, Japan) high-resolution transmission electron microscope (HRTEM). The x-ray photoelectron spectroscopy (XPS) analysis of Ce/Fe-MOF was carried out using Thermo Scientific K-Alpha. Surface functional groups were analyzed by Fourier transform infrared (FT-IR) spectroscopy (670 IR spectrometer). Cyclic voltammetry testing was performed using an electrochemical analyzer (CHI 760E workstation). Electron paramagnetic resonance (EPR) spectra were recorded using a Bruker EMX nanospectrometer. Absorption spectra were determined using a visible absorption spectrophotometer T2602 from Yoke Instruments (Shanghai, China), and the oxidized TMB was measured at 652 nm using UV spectrophotometry. The redox behavior of Ce/Fe-MOF was verified by cyclic voltammetry (CV). Cyclic voltammetry (CV) was performed using an electrochemical analyzer (CHI 760E workstation) with Ag/AgCl electrode and Pt sheet as the reference electrode and auxiliary electrode, respectively. The glassy carbon electrodes (GCE) modified with Ce/Fe-MOF, Ce-MOF and Fe-MOF were used as working electrodes for cyclic voltammetry in the potential range of −0.3 ∼ 1.1 V.

### 2.3 Preparation of bimetallic Ce/Fe-MOF

**Fig. 1:**
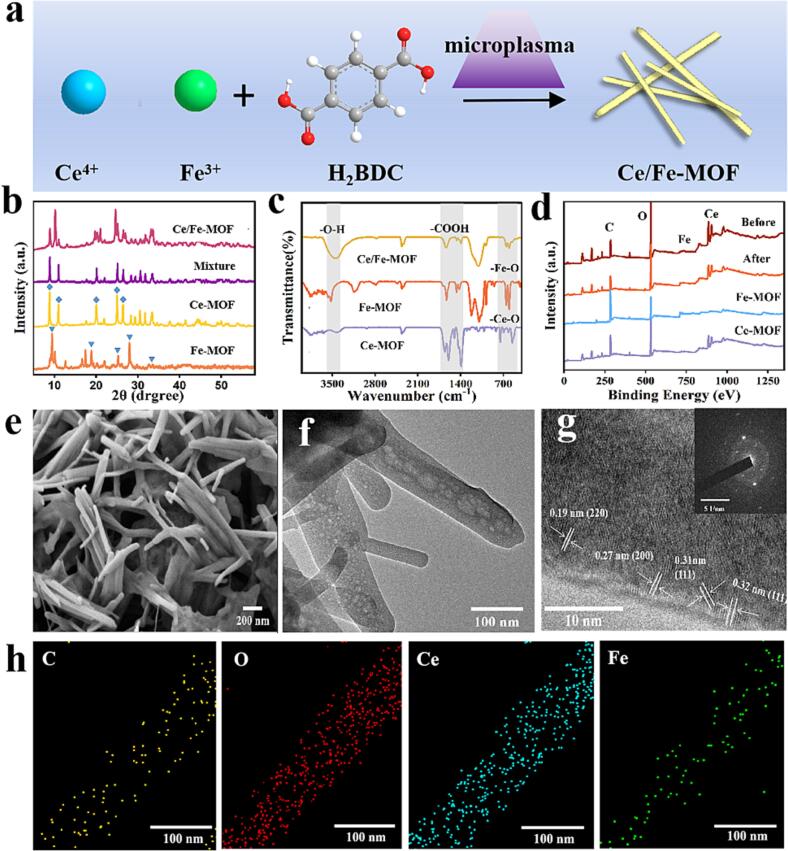
(a) The schematic diagram showing synthesis of Ce/Fe-MOF. (b) XRD patterns of Ce/Fe-MOF, Ce-MOF, Fe-MOF, and the mixtures of Ce-MOF and Fe-MOF. (c) FT-IR spectra of Ce/Fe-MOF, Ce-MOF and Fe-MOF. (d) XPS patterns of Ce/Fe-MOF before the reaction and after the reaction, Ce-MOF and Fe-MOF. (e) Field emission scanning electron microscopy (FE-SEM) image of Ce/Fe-MOF. (f) TEM image of Ce/Fe-MOF. (g) High-resolution TEM image of Ce/Fe-MOF (inset image: selected area electron diffraction patterns of the Ce/Fe-MOF). (h) Elemental mapping images of Ce/Fe-MOF.

Ce/Fe-MOF was synthesized in one step by dielectric barrier discharge (DBD) microplasma method with 1,3-benzenedicarboxylic acid (H_2_BDC) as ligand and Ce and Fe as metal centers (Fig. 1a). More details of the device can be found in our previous works (He et al., 2021, Xia et al., 2022, Zhou et al., 2023, Zhou et al., 2022) Briefly, 0.27 g (1 mmol) FeCl_3_⋅6H_2_O and 0.41 g (1 mmol) Ce(SO_4_)_2_⋅4H_2_O were dissolved in 10 mL DMF solution (solution A) and 0.254 g (1 mmol) H_2_BDC was dissolved in another 10 mL DMF solution (solution B).

Solution A was combined with Solution B and subsequently transferred to a dielectric barrier discharge (DBD) tube, where it underwent reaction at 80 V for 30 min. Following the reaction, the resulting precipitate was separated from the reaction mixture through centrifugation (8000 rpm for 10 min). The precipitate underwent multiple washes with ultrapure water, DMF and ethanol, and finally dried in a vacuum oven at 60 ℃ for 12 h. Ce-MOF and Fe-MOF were synthesized in the same process with 0.41 g (1 mmol) Ce(SO_4_)_2_⋅4H_2_O and 0.27 g (1 mmol) FeCl_3_⋅6H_2_O respectively.

### 2.4 Analysis of multiple enzyme-like properties of Ce/Fe-MOF

The oxidase-like properties of Ce/Fe-MOF were investigated through oxidizing TMB. 1 mL mixture of 50 μL (5 mg mL^−1^) Ce/Fe-MOF suspension, 50 μL(1 mmol) TMB and 0.9 mL(pH = 4) NaAc-HAc buffer was reacted for 6 min at room temperature, and the UV absorbance spectroscopy at 652 nm was measured. And 5 mmol, 10 mmol, and 20 mmol of p-benzoquinone (BQ), isopropyl alcohol (IPA), l-histidine (HD), and ethylenediaminetetraacetic acid (EDTA) were used as radical scavengers of O_2_•^−^, •OH, ^1^O_2_, and hole (h^+^), respectively, to explore the mechanism of oxidase-like activity of the Ce/Fe-MOF colorimetric sensor.

To explore the peroxidase properties of Ce/Fe-MOF, 1 mL mixture of 50 μL (5 mg mL^−1^) Ce/Fe-MOF suspension, 50 μL (10 mmol) H_2_O_2_, 100 μL (1 mmol) TMB and 0.8 mL NaAc-HAc buffer (pH = 4) was reacted for 6 min at room temperature, and the absorbance at 652 nm was recorded. The mechanism was explored by the same process as the above.

To study the superoxide dismutase (SOD)-like activity of Ce/Fe-MOF, the Ce/Fe-MOF was determined using the auto-oxidation method of 1,2,3-Benzenetriol, then a SOD activity assay kit was used. Superoxide anion (O_2_•^−^) is generated by xanthine and xanthine oxidase reaction system, and O_2_•^−^ can reduce nitro-blue tetrazolium to produce blue formazan, which has absorption at 560 nm. Ce/Fe-MOF acts as a superoxide dismutase to scavenge O_2_•^−^, thus inhibiting the formation of mezanine. The deeper the blue color of the reaction solution, the lower the SOD activity. Conversely, the SOD activity is higher. Xanthine was performed in the presence of xanthine oxidase in a 100 mM pH 7.4 PBS buffer solution. Nitro-blue tetrazolium solution was added and the reaction was carried out at 37 °C for 30 min. Then Ce/Fe-MOF suspensions of different concentrations were added, and after incubation for 30 min, the absorption peak of formazan at 560 nm was measured.

Some general parts relating to materials and equipment are given in the supporting information (section S1-S7).

### 2.6 Steady-State kinetics analysis

The Michaelis-Menten equation and the Lineweaver-Burk equation were used to analyze the relationship between enzyme-catalyzed reaction rate and substrate concentration (Dalziel, 1962). The Michaelis-Menten equation describes the relationship between enzyme-catalyzed reaction rate and substrate concentration, while the Lineweaver-Burk equation is commonly used for the analysis and comparison of enzyme kinetics experimental data. The Michaelis-Menten equation is shown below,

$$V=V_{\max}\times \left[S\right]/\left(K_{m}+\left[S\right]\right)$$

In the equation, *V* represents the reaction rate, *Vmax* represents the maximum reaction rate at infinite substrate concentration, *[S]* represents the substrate concentration, and *K_m_* represents the enzyme's substrate affinity constant. The Michaelis-Menten equation reveals a linear relationship between reaction rate and substrate concentration at low substrate concentrations, while the reaction rate approaches the maximum value *V_max_* as substrate concentration approaches *K_m_*. The Lineweaver-Burk equation is a linearization of the Michaelis-Menten equation, expressed as follows,

$$1/V=\left(K_{m}/V_{\max}\right)\times \left(1/\left[S\right]+1/K_{m}\right)$$

Plotting the reciprocal of the Michaelis-Menten equation yields a straight line with an intercept of 1/*V_max_* and a slope of *K_m_*/*V_max_*. The values of *K_m_* and *V_max_* can be calculated to evaluate the substrate affinity and catalytic activity of Ce/Fe-MOF (Yang et al., 2023).

### 2.7 Colorimetric detection of ascorbic acid (AA), glutathione (GSH), l-cysteine (Cys) and TAC assay

The detection of GSH, Cys, and AA relied on the color reaction between Ce/Fe-MOF and TMB. Firstly, Ce/Fe-MOF directly oxidizes colorless TMB to blue oxTMB, and then different concentrations of GSH, Cys, and AA reduce oxTMB to colorless. Thus, a simple colorimetric detection platform for GSH, Cys, and AA was established. 100 μL of oxTMB and 100 μL of different concentrations of GSH, Cys, and AA were added to an 8-well strip plate, and the absorbance was measured after a 15 min reaction at room temperature.

The practicality of the colorimetric method was further tested in food samples. Total Antioxidant Capacity (TAC) is typically defined as the millimoles of ascorbic acid (AA) in a liter of solution. AA equivalent is currently the most used and relatively accurate unit of antioxidant capacity, which can be used for various types of samples including foods, dietary supplements, and plant extracts, and can also be used to evaluate whether the antioxidant capacity of samples meets international standards (Hong et al., 2023). Therefore, representing the results of antioxidant capacity determination in AA equivalent units is a convenient, universal, accurate, and easy-to-understand approach.

Therefore, the same AA detection method was also applied to TAC detection of fresh fruits and vegetables. Fresh fruit and vegetable samples were juiced using the edible part, and the filtered supernatant is diluted 15 times and 10 times before testing respectively. The TAC of actual samples is expressed in millimoles of AA per liter (AA/L) equivalent units.

## 3 Results and discussion

### 3.1 Structural characterization of Ce/Fe-MOF

The crystal structure of Ce/Fe-MOF, Ce-MOF, Fe-MOF were determined using an X-ray diffraction (XRD) instrument (Fig. 1b). The diffraction peaks of Ce/Fe-MOF are different from those of the physical mixtures (weight ratio 1:1), while the major diffraction peaks of Ce-MOF and Fe-MOF can be found in the XRD pattern of Ce/Fe-MOF, indicating that Ce/Fe-MOF is not a simple physical mixture but a bimetallic MOF with a specific crystal structure (Liu et al., 2021). The types of functional groups in the Ce/Fe-MOF, Ce-MOF, Fe-MOF were determined by Fourier transform infrared spectroscopy (FT-IR). As shown in Fig. 1c, the peak at 3430 cm^−1^ was associated with the stretching vibration of O—H, while the characteristic peaks at 1610–1560 and 1480–1370 cm^−1^ were attributed to the asymmetric and symmetric stretching vibrations of carboxyl groups respectively, which confirms the existence of dicarboxylic acid. The infrared peak at 500–700 cm^−1^ was due to the Ce-O stretching vibration (Chen et al., 2017) and the peak at 549 cm^−1^ was attributed to the stretching vibration of Fe − O bonds (Liu et al., 2022). The characteristic peaks of Ce/Fe-MOF could be corresponded to those of single-metal MOFs, showing similar absorption peaks, indicating the successful synthesis of Ce/Fe-MOF. Surface composition was determined by X-ray photoelectron spectroscopy (XPS). Fig. 1d shows the XPS spectra of Ce/Fe-MOF, Ce-MOF and Fe-MOF, with the presence of C, O, Fe and Ce elements observed, indicating the successful synthesis of the materials. The surface morphology and microstructure of Ce/Fe-MOF were characterized by scanning electron microscopy (SEM) and transmission electron microscopy (TEM). As shown in Fig. 1e-f, Ce/Fe-MOF showed a rod-like shape, more pores and structural defects, which could provide a large surface area and catalytic active sites. In contrast, Fig. S8 displayed the SEM image of Ce-MOF, which showed its rod-shaped structure with a rough surface (Chen et al., 2018). On the other hand, Fe-MOF was found to be composed of two-dimensional nanosheets forming a three-dimensional peony-like microsphere structure. In the high-resolution TEM image of the Ce/Fe-MOF (Fig. 1g), it contains lattice stripes with spacings of 0.27 nm, 0.31 nm, and 0.19 nm, corresponding to the (2 0 0), (1 1 1), and (2 2 0) crystal planes of the Ce-MOF(Wang et al., 2019, Wang and Astruc, 2019), and the lattice spacing of 0.32 nm matches the (1 1 1) orientation of the Fe-MOF(Hu et al., 2017). As shown in the inset, the selected-area electron diffraction (SAED) images exhibit bright crystal spots, indicating that the Ce/Fe-MOF is actually crystalline, which is consistent with the XRD results. The Energy Dispersive Spectroscopy (EDS) mapping images further indicate that Fe, Ce, C, and O are uniformly distributed in the Ce/Fe-MOF (Fig. 1h).

**Fig. 2:**
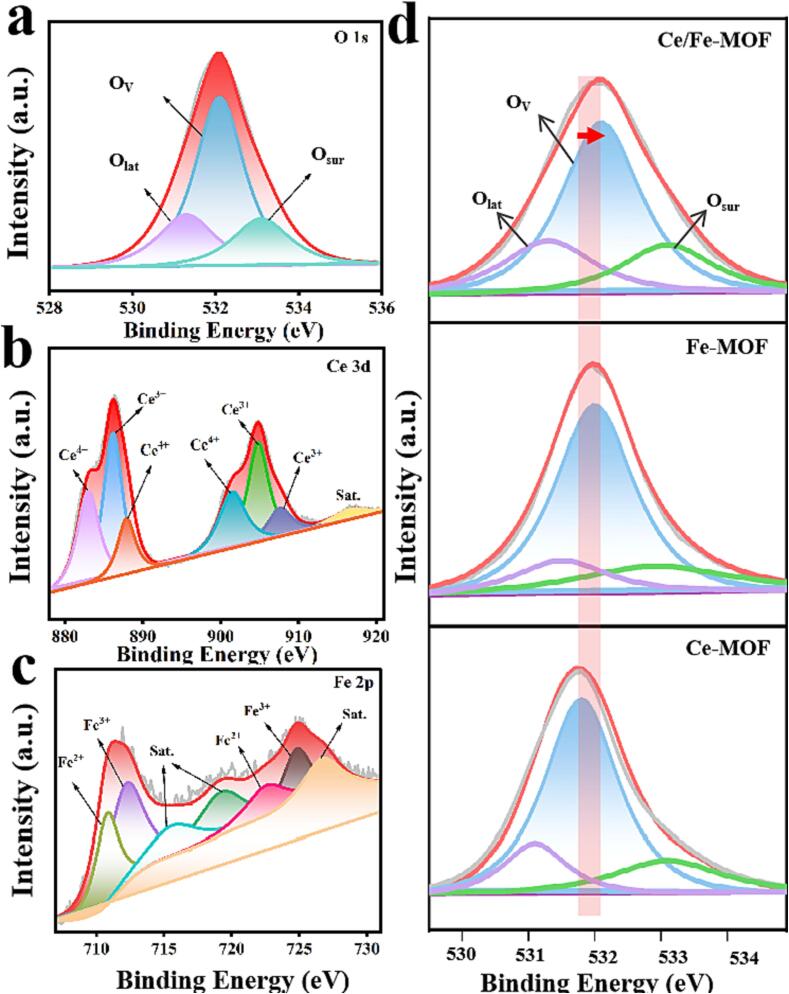
XPS patterns of Ce/Fe-MOF, (a) O 1 s, (b) Ce 3d, (c) Fe 2p. (d) XPS spectra of O 1 s of Ce/Fe-MOF, Fe-MOF and Ce-MOF.

The oxygen vacancy is an indicator of lattice defect degree, and lattice defects are usually generated by the reduction of high-valent metal ions to low-valent metal ions (Guo et al., 2020). The chemical states of Ce/Fe-MOF were analyzed by X-ray photoelectron spectroscopy (XPS). As shown in Fig. 2a, The O (1 s) spectrum can be fitted with three binding energy (BE) peaks, corresponding to lattice oxygen (O_lat_), surface adsorption oxygen (O_sur_), and defect oxygen (O_v_) (Mu et al., 2021). The presence of mixed valence states of the elements further confirms the existence of oxygen vacancies. As shown in Fig. 2b, XPS spectra of Ce 3d indicate the coexistence of mixed valence states (Ce^3+^/Ce^4+^) in the material, with peaks associated with Ce^4+^ located at 883.2, 887.8, 901.4, and 907.8 eV, and peaks associated with Ce^3+^ located at 886.1 and 904.9 eV, respectively. In addition, a characteristic satellite peak at 917.4 eV further confirms the presence of Ce^3+^ species (Xiong et al., 2015). Fig. 2c illustrates the Fe 2p XPS spectrum, there are two main peaks at 711.1 and 725.1 eV, which correspond to the Fe 2p3/2 and 2p1/2 levels, respectively. The binding energies of Fe 2p1/2 and Fe 2p3/2 are 707.9 and 724.8 eV, respectively, while those of Fe^2+^ are 710.8 and 722.6 eV for Fe 2p1/2. Additionally, characteristic satellite peaks at 715.6, 719.2, and 726.7 eV are observed (Chen et al., 2019), further indicating the presence of Fe^3+^ species. Moreover, the coexistence of mixed valence states (Ce^3+^/Ce^4+^, Fe^2+^/Fe^3+^), also in Ce-MOF and Fe-MOF respectively (Fig. S9). Fig. 2d showed the O 1 s spectra of Ce/Fe-MOF, Ce-MOF, and Fe-MOF. Compared to the single-metal MOFs, the binding energy position of the oxygen peak of Ce/Fe-MOF significantly shifts towards higher binding energy, indicating a change in the electronic structure of the oxygen element and the possible existence of oxygen vacancies(Zhu et al., 2017). In addition, it was confirmed by electron paramagnetic resonance (EPR) spectroscopy that oxygen vacancies exist in the Ce/Fe-MOF (Fig. S10). Meanwhile, before the reaction of Ce/Fe-MOF with TMB, the Fe^3+^/Fe^2+^ ratio was approximately 1.49 and the Ce^4+^/Ce^3+^ ratio was approximately 1.18. After the reaction, the Fe^3+^/Fe^2+^ ratio decreased to 0.94 whereas the Ce^4+^/Ce^3+^ ratio increased to approximately 1.27. These results suggest that both iron (Fe) and cerium (Ce) elements function as active sites in the catalytic reaction, working synergistically to enhance catalytic activity (Table S1).

### 3.2 Multiple enzyme-like properties of Ce/Fe-MOF

**Fig. 3:**
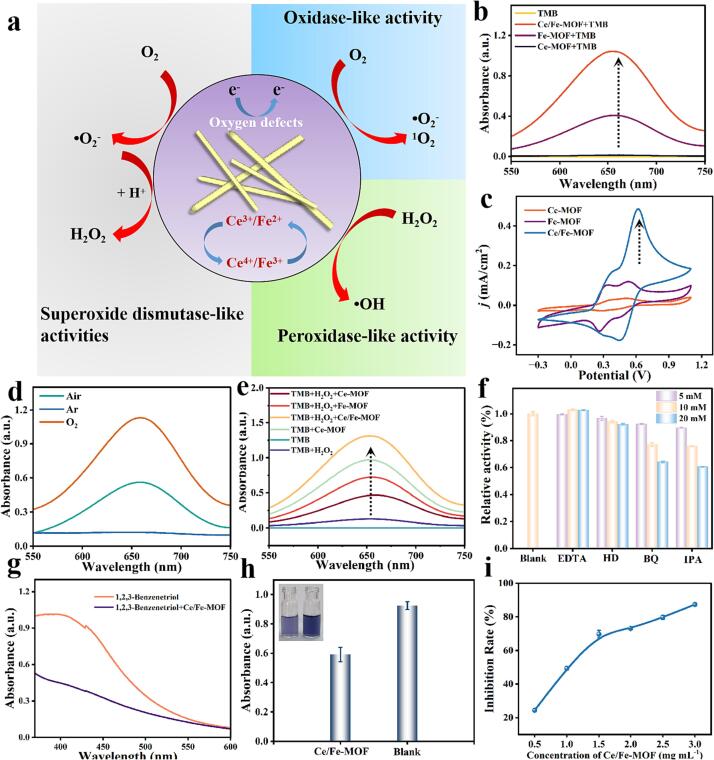
(a) Schematic diagram of multiple enzyme-like properties of Ce/Fe-MOF. (b) The feasibility of oxidase-like activity of Ce/Fe-MOF. (c) Cyclic voltammograms of Ce/Fe-MOF, Ce-MOF and Fe-MOF modified glassy carbon electrodes; buffer solution: HAc-NaAc. (d) UV absorbance curves of the systems under different gas conditions: O_2_, Air and Ar. (e) The feasibility of peroxidase-like activity of Ce/Fe-MOF. (f) Free radical capture ability of Ce/Fe-MOF. (g) Autoxidation of 1,2,3-benzenetriol in the presence and absence of Ce/Fe-MOF. (h) Exploration of the feasibility of superoxide dismutase of Ce/Fe-MOF using SOD activity assay kit. (inset image: photographs of color changes) (i) The percentage inhibition of O_2_•^−^.

The catalytic performance of Ce/Fe-MOF is crucial for highly sensitive sensing of TAC. Ce/Fe-MOF exhibits excellent catalytic performance due to the abundant oxygen vacancy and collaboration between iron and cerium. The synergistic interaction between iron and cerium not only enhances the efficiency of the catalyst but also imparts the Ce/Fe-MOF with multiple enzymatic properties, including peroxidase-like, oxidase-like, and superoxide dismutase mimetic activities (Fig. 3a).

#### 3.2.1 Oxidase-like activity of Ce/Fe-MOF

The evaluation of oxidase-like activity using TMB as a substrate is a widely used method to assess catalytic activity, typically relying on measuring the absorbance change in the reaction system. As shown in Fig. 3b, with Ce-MOF, the absorption at 652 nm (representing the blue oxidized form of TMB) is almost negligible. For Fe-MOF, there is a weak absorption peak at 652 nm. However, with Ce/Fe-MOF, a strong absorption peak appeared at 652 nm, suggesting that Ce/Fe-MOF exhibits significant catalytic activity towards the oxidation of TMB. The catalytic efficiency and affinity of Ce/Fe-MOF were evaluated by measuring its kinetic constants. Fig. S11 shows that the *K_m_* and *V_max_* values of Ce/Fe-MOF for TMB are 0.62 mM and 49.1 × 10^−8^ M s^−1^, respectively. In contrast, Ce-MOF does not exhibit oxidase-like activity, and the *K_m_* and *V_max_* values of Fe-MOF are 1.79 mM and 34.5 × 10^−8^ M s^−1^, respectively (Fig. S12). The lower *K_m_* value observed for Ce/Fe-MOF indicates a stronger affinity for the substrate TMB, On the other hand, the larger *V_max_* (maximum reaction velocity) value indicates a higher conversion rate from the substrate (TMB) to the product, demonstrating a higher oxidase-like activity of Ce/Fe-MOF (Table S2). The oxidation–reduction behavior of Ce/Fe-MOF was explored by cyclic voltammetry (CV). As can be seen in Fig. 3c, a significant oxidation–reduction peak appeared for Ce/Fe-MOF than Fe-MOF and Ce-MOF, which indicates that the electron transfer efficiency in the oxidation–reduction couple of Ce/Fe-MOF is superior to that of Fe-MOF and Ce-MOF. To ascertain whether the oxidative enzyme-like activity of Ce/Fe-MOF is contingent on oxygen, a series of gas-dependent experiments were conducted in air, oxygen, and nitrogen environments. Fig. 3d shows that the absorbance value is highest under oxygen-rich conditions, demonstrating that oxygen is one of the main factors affecting the oxidative enzyme-mimicking activity of Ce/Fe-MOF. Furthermore, free radical capture experiments were conducted to explore the free radicals in the reaction system, and the results showed that O_2_•^−^, •OH and ^1^O_2_ played important roles in the catalytic process (Fig. S13).

#### 3.2.2 Peroxidase-like activity of Ce/Fe-MOF

The peroxidase-like activity of Ce/Fe-MOF was verified using hydrogen peroxide and TMB substrate. In Fig. 3e, the results showed that in the presence of hydrogen peroxide, a higher absorbance spectroscopy with Ce/Fe-MOF, indicating it has enhanced peroxidase-like activity. As shown in Fig. 3f, in free radical capture experiments, it was found that •OH, O_2_•^−^, and ^1^O_2_ all played a role in the catalytic process, and •OH had the greatest effect. The kinetic constants of Ce/Fe-MOF was shown in Fig. S14 a-d, the reaction rate gradually increased with increasing hydrogen peroxide concentration, and the double reciprocal plot showed that the *K_m_* value of Ce/Fe-MOF was 1.67 mM, and the *V_max_* value was 17.7 × 10^−8^ M s^−1^. Similarly, when the hydrogen peroxide concentration was 4 mM, the enzyme kinetic parameters of Ce/Fe-MOF for TMB were determined. The *K_m_* value of Ce/Fe-MOF was 2.02 mM, and the *V_max_* value was 7.94 × 10^−8^ M s^−1^, indicating good peroxidase-like activity. Compared with Ce-MOF, Fe-MOF and other Ce-based and Fe-based nanozymes, Ce/Fe-MOF exhibits excellent kinetic parameters for both oxidase-like and peroxidase-like activity, as shown in Table 1.

**Table 1:** Kinetic parameters of oxidase-like enzymes and peroxidase-like enzymes.

	Nanozymes	*K_m_* (TMB)/mM		*V_max_* (TMB)/10^−8^M·s^−1^		Ref.
Oxidase-like activity	ZIF-67	13.60		0.35		(Wang et al., 2018, Wang et al., 2018)
	MVCM-DBD	1.31		24.70		(Xia et al., 2022)
	Fe-N/C	0.94		59.80		(Chen et al., 2020, Chen et al., 2020)
	Cu-MOF	4.11		55.50		(Wang et al., 2018, Wang et al., 2018)
	MIL-53(Fe)	1.08		8.78		(Ai et al., 2013)
	**Ce/Fe-MOF**	**0.62**		**49.1**		**This work**
		***K_m_*/mM**		***V_max_*/10**^−^**^8^M·s^−1^**		
Peroxidase-like activity		**TMB**	**H_2_O_2_**	**TMB**	**H_2_O_2_**	
	FeNi MOF	1.97	0.01	1.31	5.29	(Wang et al., 2020, Wang et al., 2020)
	Fe-MOF-GOx	2.60	1.30	5.60	2.50	(Xu et al., 2019)
	MIL-53(Fe)	1.08	0.04	8.78	1.86	(Ai et al., 2013)
	Fe—N—C SAzymes	3.60	12.2	11.6	35.6	(Jiao et al., 2019)
	**Ce/Fe-MOF**	**1.67**	**2.02**	**17.7**	**7.94**	**This work**

#### 3.2.3 Superoxide dismutase-like activity of Ce/Fe-MOF

First, the superoxide scavenging ability of Ce/Fe-MOF was determined using the auto-oxidation method of 1,2,3-Benzenetriol (Guo et al., 2022). Fig. 3g shows that in the presence of Ce/Fe-MOF, the autoxidation of 1,2,3-Benzenetriol is inhibited, indicating that Ce/Fe-MOF possesses superoxide dismutase (SOD)-like activity. The SOD-like activity was further verified using a SOD activity assay kit. Fig. 3h illustrates the percentage inhibition of formazan. As shown in Fig. 3i, Ce/Fe-MOF can effectively eliminate O_2_•^−^, thereby inhibiting the formation of formazan. The above results indicate that Ce/Fe-MOF has a good inhibitory effect on superoxide radicals and can effectively clear O_2_•^−^.

### 3.3 Colorimetric detection of AA, GSH, Cys

AA, GSH, and Cys are common antioxidants that can be detected through colorimetric assays by reduction of oxTMB and monitoring its UV absorbance change, then assess the antioxidant capacity of antioxidants. Based on the color response between Ce/Fe-MOF and TMB, a simple method was developed as a sensing platform for the quantitative detection of AA, GSH, and Cys. Here, we mainly discuss that AA, GSH, and Cys also have sensing effects (supplementary section S-3 and S-4).

#### 3.3.1 Colorimetric detection of AA

**Fig. 4:**
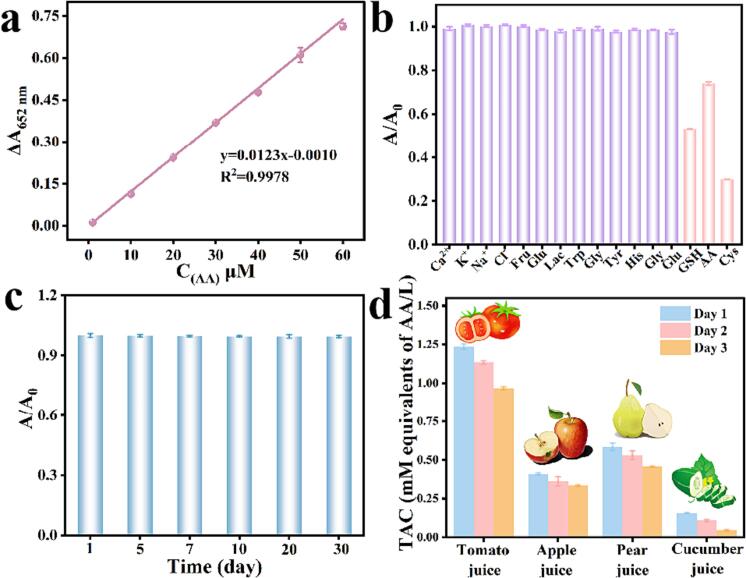
(a) Linear curves of the absorbance versus concentration of AA. (b) Interference test for AA. (c) Stability of Ce/Fe-MOF over different numbers of days, tested with AA. (d) TAC content of fresh apples, pear, cucumbers, and tomato in different time.

As a ubiquitous antioxidant, ascorbic acid (AA), also known as vitamin C and found abundantly in fresh fruits and vegetables, plays a vital role in mitigating oxidative stress within cells. This is achieved by its ability to react with reactive oxygen species (ROS) through redox reactions, thereby safeguarding cells from oxidative damage. Ce/Fe-MOF catalyzes the oxidation of substrate TMB and produces colored product oxTMB. When AA is present in the system, it can reduce the oxidation reaction of TMB as an antioxidant, leading to a decrease in absorbance value. By measuring the change in absorbance value, the concentration of AA can be reflected, thus achieving quantitative detection of AA. As shown in Fig. 4a, in the range of 2–60 μM of AA, the absorbance change of TMB at 652 nm shows a good linear relationship of y = 0.0123x-0.0010 and a correlation coefficient (R^2^) of 0.9978. The detection limit (LOD) of AA was calculated using the formula LOD = 3σ/k (σ is the standard deviation of the blank signal measured 11 times, and k is the slope of the standard curve), which was 1.3 μM. It indicates that this Ce/Fe-MOF method displayed a relative high-level of sensitivity when compared with various other colorimetric methods, as presented in Table S3.

#### 3.3.2 Anti-interference ability and stability measurement

To test the anti-interference ability of the colorimetric sensor, potential interfering substances were tested in real samples, including 100-fold concentrations of K^+^, Na^+^, Ca^2+^, Cl^−^, glucose (Glu), fructose (Fru), α-Lactose (Fru), glycine (Gly), glutamic acid (Glu), l-histidine (His), l-tryptophan (L-Tr), glutamine (l-Glu), and tyrosine (L-Ty). As shown in Fig. 4b, except for Cys and GSH, there was almost no significant inhibitory effect on AA compared to interference. Considering that the concentration of amino acids (GSH and Cys) in foods is much lower than that of AA, the experimental results show that the method can be applied to the practical detection of TAC.

Good stability is crucial for the practical application of colorimetric sensing methods. The analytical performance of the same Ce/Fe-MOF material was compared after different days of storage, and the results showed that the performance remained almost unchanged after 30 days of storage, indicating the stability of the Ce/Fe-MOF catalyst and its potential application for the detection of agricultural products (Fig. 4c).

### 3.6 TAC assay

The accuracy and reliability of the Ce/Fe-MOF colorimetric assay for Total Antioxidant Capacity (TAC) evaluation were verified using the standard addition method, as presented in Table 2. Fresh apple juice, pear juice, cucumber juice was initially diluted 10 times, and tomato juice were initially diluted 15 times, respectively (Juice was prepared and stored at 2–8 °C). Real samples of ascorbic acid (AA) with known concentrations were then added to these diluted samples. By comparing the TAC content detected using the standard curve method with the TAC content obtained after deducting the added known concentrations of AA, the recovery rate of AA in the real samples was found to range from 91 % to 107 %. Additionally, the relative standard deviation (RSD) of the results was found to be less than 1.38 %. These results demonstrate that the Ce/Fe-MOF colorimetric assay is reliable and feasible for accurately detecting TAC in real samples. The use of the standard addition method helps confirm the accuracy of the assay by validating its ability to recover known concentrations of AA added to the samples, and the AA concentrations detected by the Ce/Fe-MOF colorimetric assay were comparable to the test results using TAC assay kits (Fig. S15) and Trolox/ORAC values (Table S4 in SI). Moreover, the changes in TAC of the various samples were analyzed over different storage days. The TAC of fresh fruit was readily monitored by our proposed assessment system with varied storage time, showing a gradually decreasing trend (as shown in Fig. 4d). Overall, the study demonstrates the reliability of the Ce/Fe-MOF colorimetric assay for TAC evaluation in real samples and provides valuable insights into the changes in TAC during the storage of fresh fruits. This information can be significant for assessing the nutritional value and quality of fruits over time.

**Table 2:** The results of TAC determination in the four samples and the recoveries of AA in the corresponding samples.

Sample	TAC (μM)	RSD (%)	AA added (μM)	Equivalent AA found (μM)	RSD (%)	AA recovery (%)
Tomato juice	41.17	1.38	5.00	46.29	0.99	102
			10.00	50.71	1.31	95
			15.00	55.65	0.55	97
Cucumber juice	7.76	2.67	5.00	12.93	2.09	103
			10.00	18.35	1.34	106
			15.00	23.10	1.87	102
Pear Juice	29.61	2.68	5.00	34.53	1.32	98
			10.00	39.95	1.36	103
			15.00	44.86	1.24	102
Apple Juice	20.41	1.61	5.00	25.23	1.81	96
			10.00	29.54	1.66	91
			15.00	36.42	1.74	107

## 4 Conclusion

In summary, this study successfully synthesized highly active Ce/Fe-MOF nanozyme using microplasma synthesis. The nanozyme exhibited unique enzymatic properties, including peroxidase-like, oxidase-like, and superoxide dismutase-like activities. A colorimetric sensing method for the quantification of total antioxidant capacity (TAC) was established utilizing the color reaction between Ce/Fe-MOF and TMB, which had short analysis time of 15 min, good linear range of 2–60 μM and low detection limit of 1.3 μM. It was successfully used for TAC detection of fruits and vegetables with good stability, accuracy and reliability. Overall, the study's outcomes underscore the importance of nanozyme technology in advancing antioxidant detection and the potential of the Ce/Fe-MOF colorimetric assay for future applications in food industries, sensing devices, and life science research endeavors. These advancements have implications for improving human health and promoting sustainable practices in the food sector.

## CRediT authorship contribution statement

**Yi Xia:** Writing – review & editing, Writing – original draft, Visualization, Validation, Methodology, Investigation, Formal analysis, Data curation, Conceptualization. **Juan He:** Writing – review & editing, Writing – original draft, Validation, Software, Methodology, Investigation, Formal analysis, Data curation, Conceptualization. **Long Tang:** Writing – review & editing, Visualization, Validation, Resources, Methodology, Investigation, Conceptualization. **Miao Hu:** Writing – review & editing, Validation. **Jie Zhou:** Writing – review & editing, Visualization, Software, Resources, Data curation. **Yao-Yu Xiao:** Writing – review & editing, Supervision, Resources, Project administration, Investigation, Funding acquisition. **Zhi-Chao Jiang:** Writing – review & editing, Writing – original draft, Visualization, Validation, Supervision, Software, Resources, Project administration, Methodology, Funding acquisition, Formal analysis, Conceptualization. **Xue Jiang:** .

## Declaration of competing interest

The authors declare that they have no known competing financial interests or personal relationships that could have appeared to influence the work reported in this paper.

## Data Availability

No data was used for the research described in the article.
