# Supplementary material for: Multifunctional bimetallic MOF with oxygen vacancy synthesized by microplasma for rapid total antioxidant capacity assessment in agricultural products
Source: Food Chem X. 2024 Feb 20;21:101247. doi: 10.1016/j.fochx.2024.101247 (PMC10907182; doi:10.1016/j.fochx.2024.101247)
Supplement: Supplementary data 1 [file mmc1.docx]

**Supplementary Information**

**Multifunctional bimetallic MOF with oxygen vacancy synthesized by microplasma for rapid total antioxidant capacity assessment in agricultural products**

Yi Xia^1#^, Juan He^1#^, Long Tang^1^, Miao Hu^1^, Jie Zhou^1^, Yao-Yu Xiao^2, 3^, Zhi-Chao Jiang^2, 3^*, Xue Jiang^1^*

^1^ Key Laboratory of Land Resources Evaluation and Monitoring in Southwest, Ministry of Education, College of Chemistry and Materials Science, Sichuan Normal University, Chengdu, 610068, China

^2^ School of Mechanical Engineering, Sichuan University, Chengdu, 610065, China

^3^ State Key Laboratory of Polymer Materials Engineering, Sichuan University, Chengdu, 610065, China

^#^ Yi Xia and Juan He contributed equally to this study and share first authorship.

*E-mail: jiangxue.2020@tsinghua.org.cn (X. J.); zhichao.jiang@scu.edu.cn (Z. -C. J.)

**Table of Contents**

| **Section** | **Content** | **Page No.** |
| --- | --- | --- |
| **S-1** | **Schematic diagram of microplasma experiment device** | **3** |
| **S-2** | **Optimization of synthesis conditions** | **4** |
| **S-3** | **Oxidase-like catalytic properties of Ce/Fe-MOF** | **5-7** |
| **S-4** | **Colorimetric detection of GSH** | **8-9** |
| **S-5** | **Colorimetric detection of Cys** | **10** |
| **S-6** | **Supplementary Figures (S7-S15)** | **11-18** |
| **S-7** | **Supplementary Tables (S1-S4)** | **19-22** |

**Section S-1: Schematic diagram of microplasma experiment device**

A simple and portable dielectric barrier discharge (DBD) reactor was designed (Scheme S1), which was composed of an open concentric glass cylinder tube (reactants in the outer cylinder), a copper rod and a copper wire, where the copper rod covered with carbon cloth was in the inner cylinder, the copper wire was wrapped outside the tube, and the copper both were connected to an electrical power supply. Bright blue plasma was observed in the reactants when the power was on.


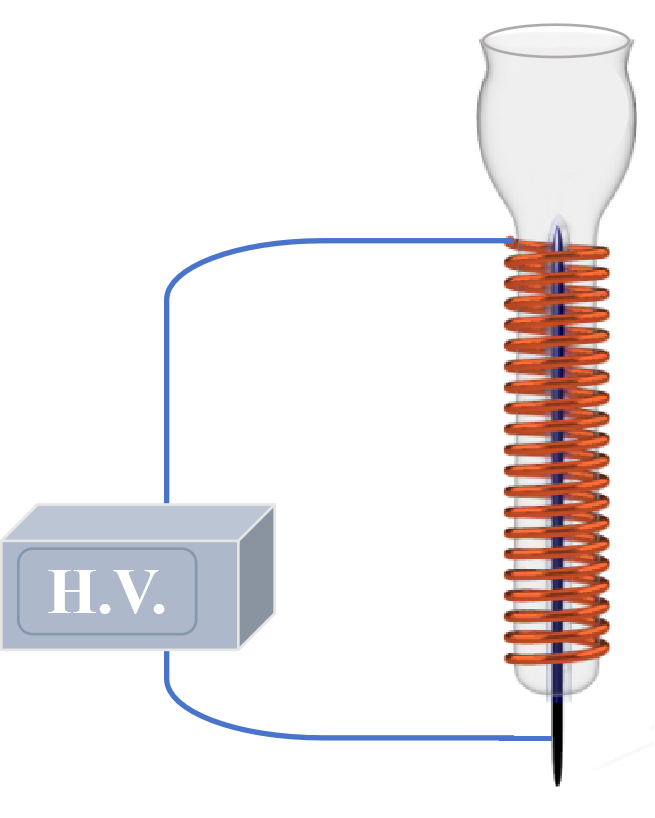


Fig.S1 Schematic of the DBD-based reactor involving liquid-phase plasma.

**Section S-2: Optimization of synthesis conditions**

The catalytic activity of different molar ratios of iron and cerium on the chromogenic substrates was first optimized, and Fig. S1 shows that the material exhibited the highest catalytic activity when the incorporated content ratio was 1:1, therefore, the catalyst with a molar ratio of cerium to iron of 1 was used in the following experiments.


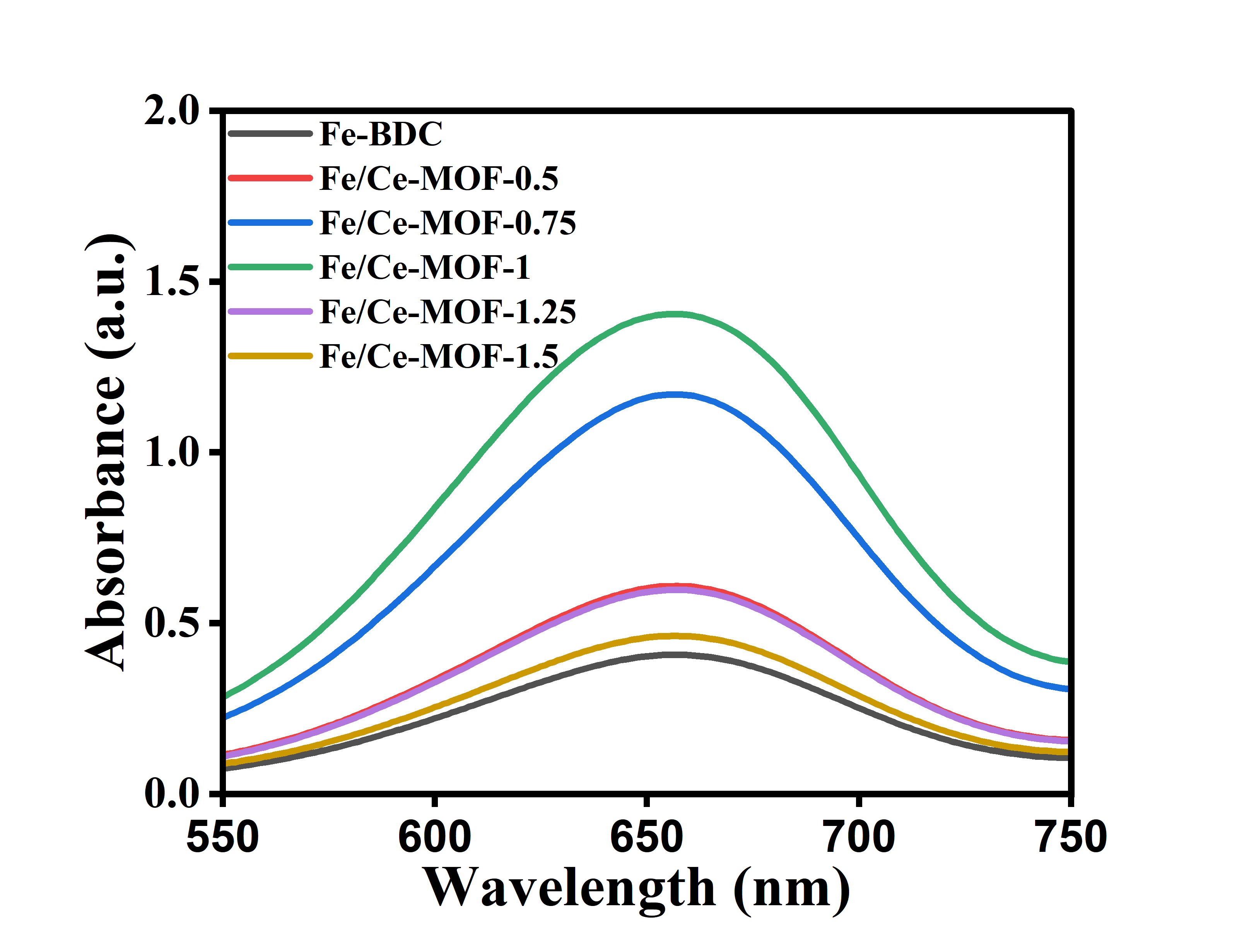


Fig.S2 Optimization of synthesis conditions.

**Section S-3: Oxidase-like catalytic properties of Ce/Fe-MOF**

Optimizing the conditions for oxidase-like activity typically involves studying multiple experimental parameters, including pH, reaction time, catalyst dosage, and TMB concentration. Experimental results have shown that these conditions can significantly impact the catalytic activity and efficiency of oxidase-like activity. First, pH plays a crucial role in oxidase-like activity, and by conducting catalytic reactions at different pH values, we observed different activities of oxidase-like activity at different pH values. As shown in Fig. S2, Ce/Fe-MOF exhibited the best catalytic performance at a pH of 4 in the buffer solution, so subsequent experiments were carried out under the conditions of NaAc-HAc buffer solution (0.1 M, pH 4).


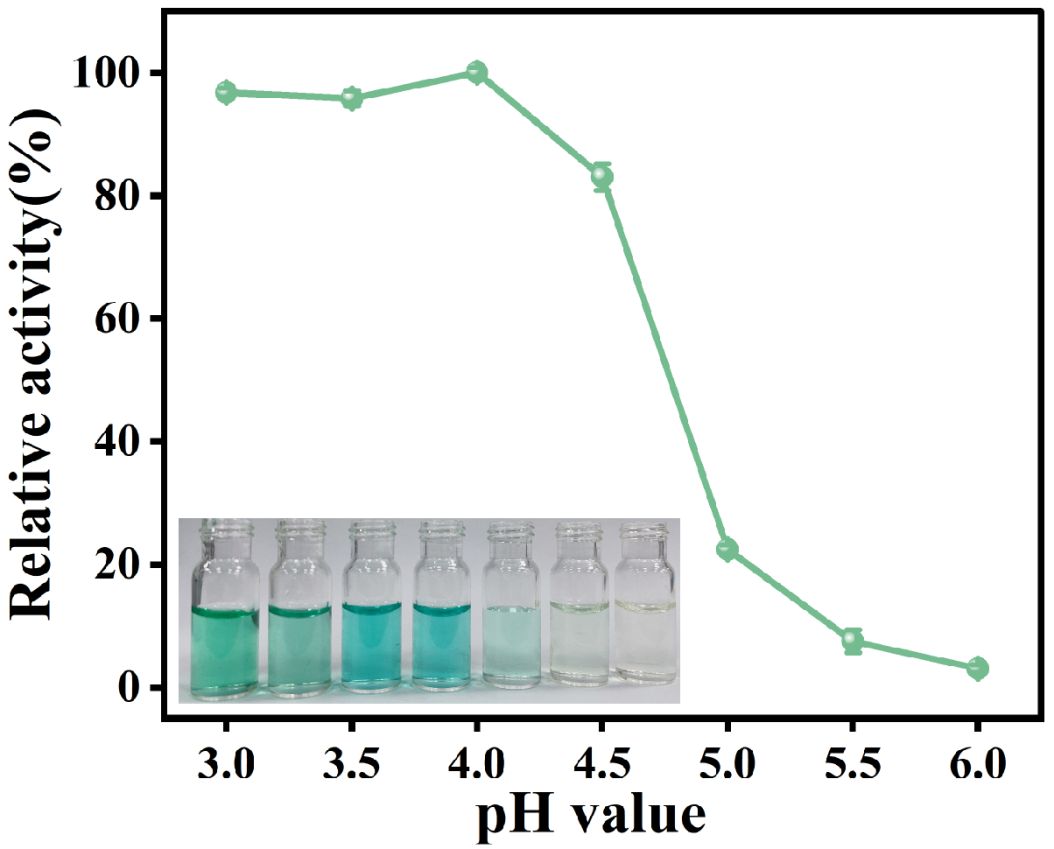


Fig. S3 Optimization of the pH.

Secondly, the length of the catalytic reaction time directly affects the evaluation results of oxidase-like activity. Shorter reaction times may not fully demonstrate the catalytic ability of the oxidase-like enzyme, while longer reaction times may result in overreaction, affecting the accuracy of the results. By conducting experiments at different catalytic reaction times, as shown in Fig. S3, it was observed that the solution began to change from blue to green and yellow after a reaction time of 6 min. This may be due to the fact that with increasing reaction time, oxTMB further catalyzes oxidation to form diamine (yellow product). Therefore, a reaction time of 6 min is the optimal time for this catalytic reaction system.


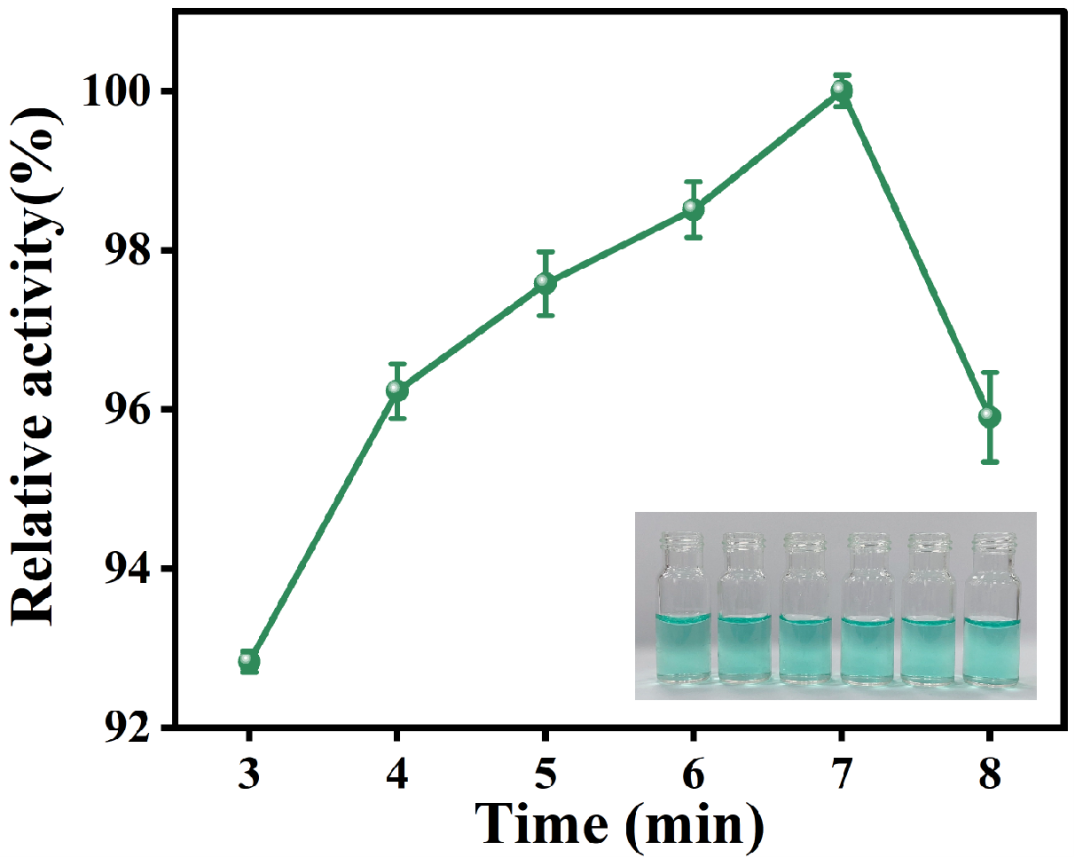


Fig. S4 Optimization of the time.

In addition, the amount of catalyst used is also a key factor affecting the activity of the class oxidase. Insufficient catalyst dosage may result in a low reaction rate, while excessive catalyst dosage may cause the catalyst to be excessively activated, thereby interfering with the reaction products or damaging the catalytic ability of the nanoenzyme. Therefore, the experimental results showed that when the catalyst concentration reached 0.5 mg/mL, the catalytic effect was optimal. With the increasing concentration of the catalyst, the color of the solution gradually changed from blue to green and yellow, possibly due to the further oxidation of oxTMB catalyzed by the increasing concentration of the catalyst to form diamine (yellow product), so the catalyst concentration in the subsequent experiments was kept at 0.5 mg/mL (Fig. S4)


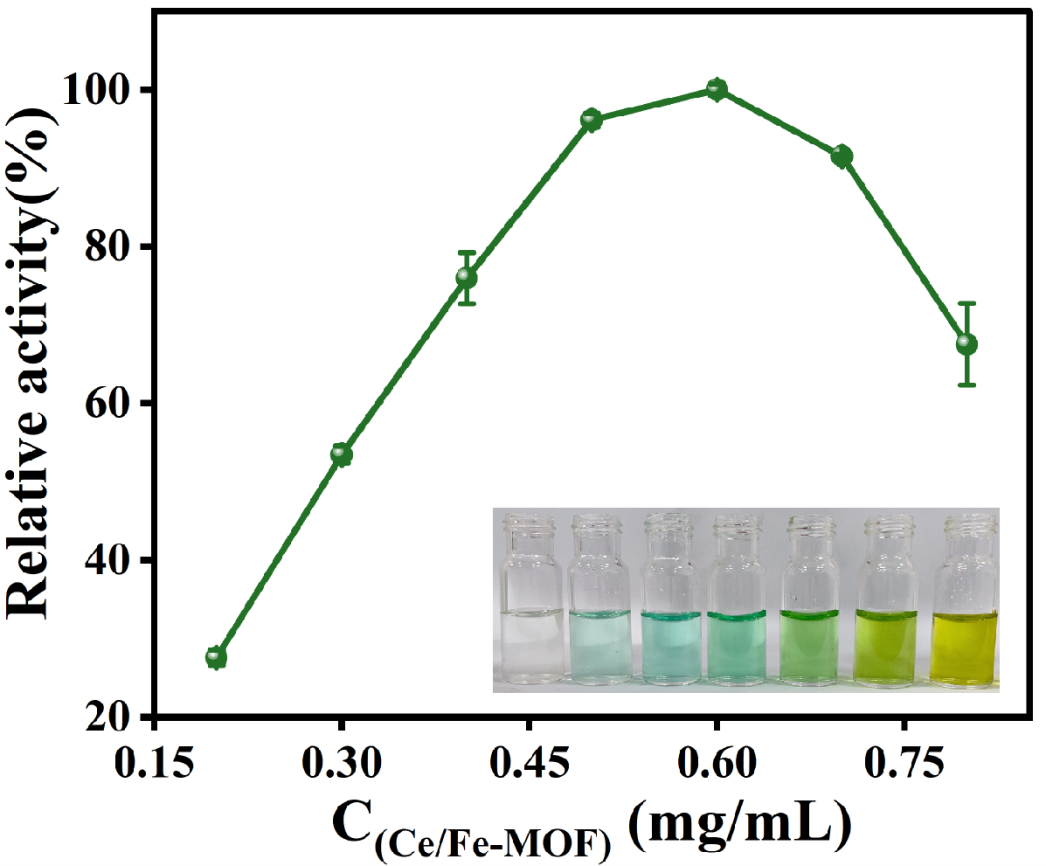


Fig. S5 Optimization of the concentration of Ce/Fe-MOF.

**Section S-4:** **Colorimetric detection of GSH**

GSH is an important antioxidant in the body and plays a crucial role in maintaining homeostasis. Traditional GSH detection methods rely mainly on high-performance liquid chromatography and other specialized instruments, which are complex to operate and have high costs, limiting their practical application. However, the GSH detection method based on oxidase-like nanozyme has the advantages of simplicity, rapidity, high sensitivity, and strong specificity, and can realize fast quantitative detection of GSH. Based on the enzymatic activity of Ce/Fe-MOF, TMB can be used for quantitative detection of GSH. Based on the catalytic activity of Ce/Fe-MOF as a mimetic oxidase, the colorless TMB is converted into blue ox TMB. Upon addition of GSH, the color of the solution changes and the absorbance decreases, which shows a linear relationship with the concentration of GSH. By measuring the absorbance of the solution after the reaction, the concentration of GSH in the sample can be calculated. The experimental results show that in the colorimetric detection system of GSH, the linear relationship between absorbance and GSH concentration is y=0.0108x+0.0198 in the concentration range of 2-50 μM, and the correlation coefficient (R^2^) is 0.9992. The detection limit (LOD) of GSH is 1.2 μM (Fig.S5)


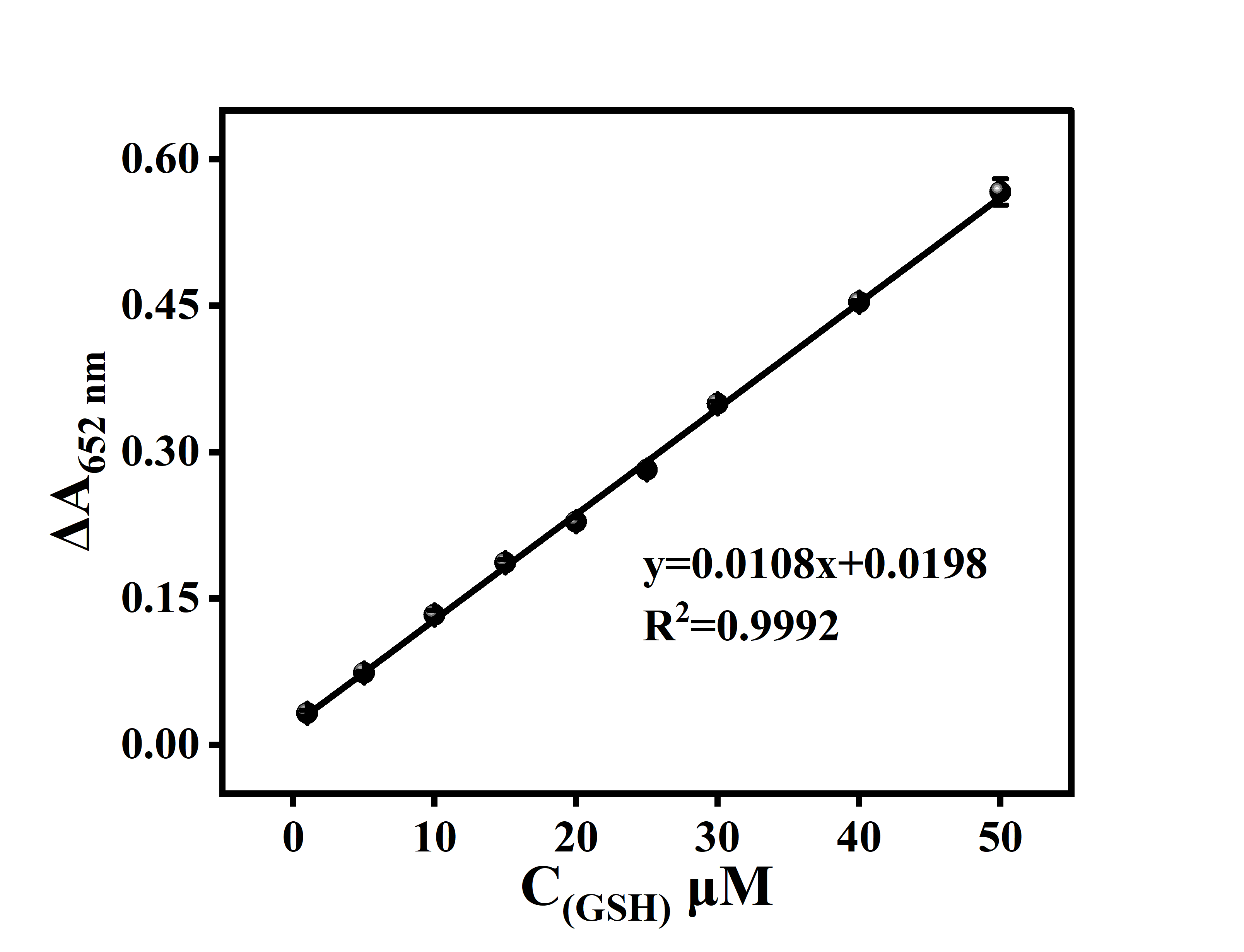


Fig. S6 Linear curves of the absorbance versus concentration of GSH.

**Section S-5:** **Colorimetric detection of Cys**

L-Cysteine (Cys) is an important amino acid that plays various biological roles in the human body, such as participating in protein synthesis, cell proliferation and differentiation, and regulating the immune system. Additionally, Cys is an important antioxidant that can work in synergy with other antioxidants such as glutathione to protect the body from oxidative stress. Similar to the GSH detection, in the Cys colorimetric detection system, the absorbance is linearly related to the Cys concentration with a linear range of 2-30 μM and a correlation coefficient (R^2^) of 0.9957. The detection limit (LOD) for Cys is 1.4 μM (Fig.S6)


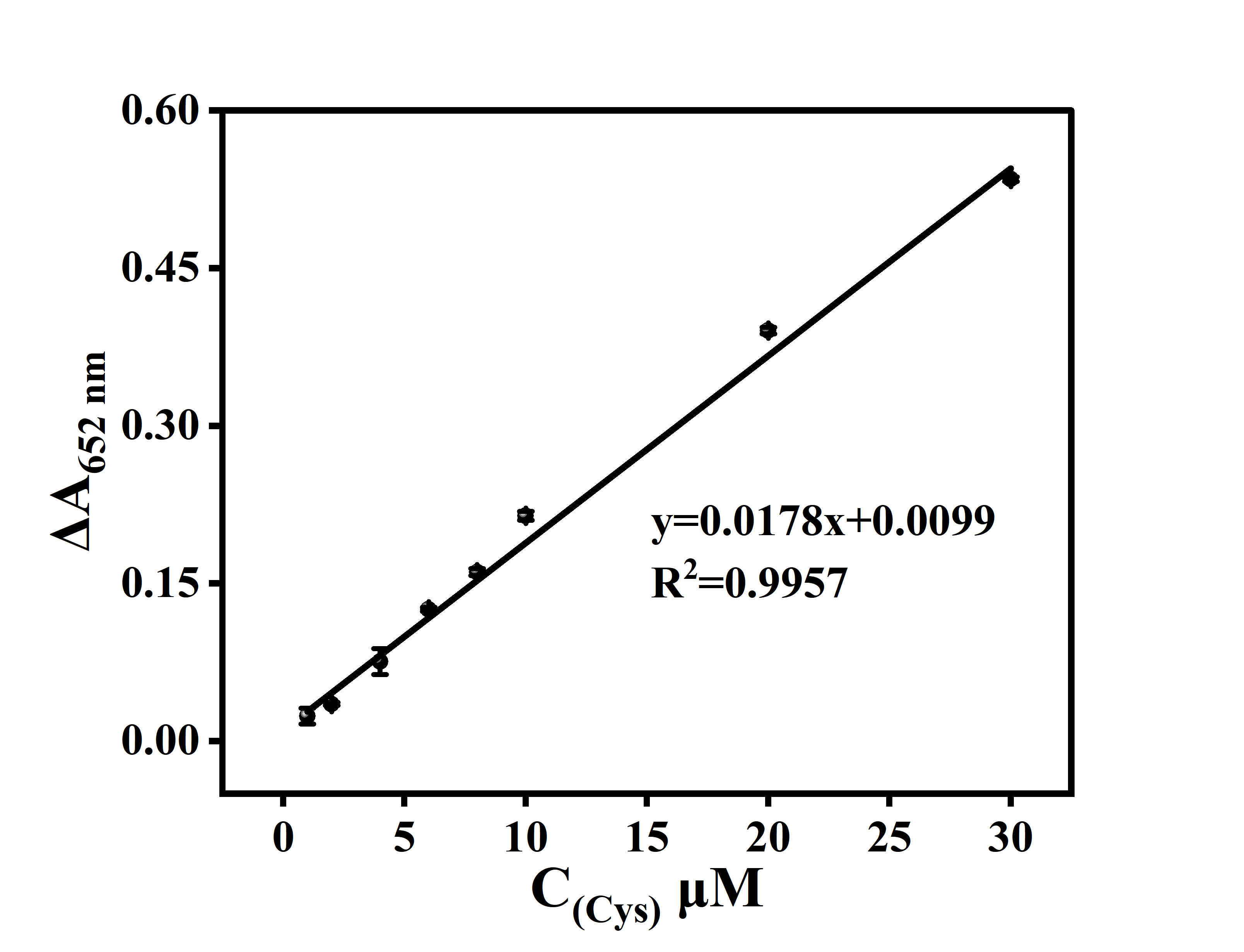


Fig. S7 Linear curves of the absorbance versus concentration of Cys.

**Section S-6: Supplementary Figures (S8-S15)**


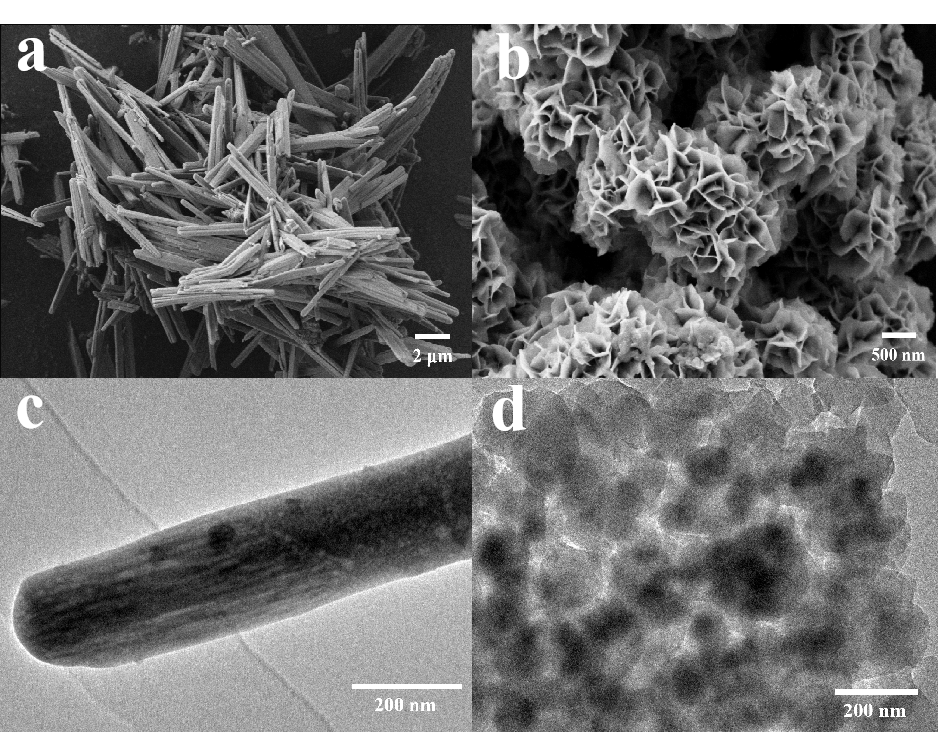


Fig. S8 FESEM image of Ce-MOF (a) and Fe-MOF (b). TEM image of Ce-MOF (c)and Fe-MOF (d).


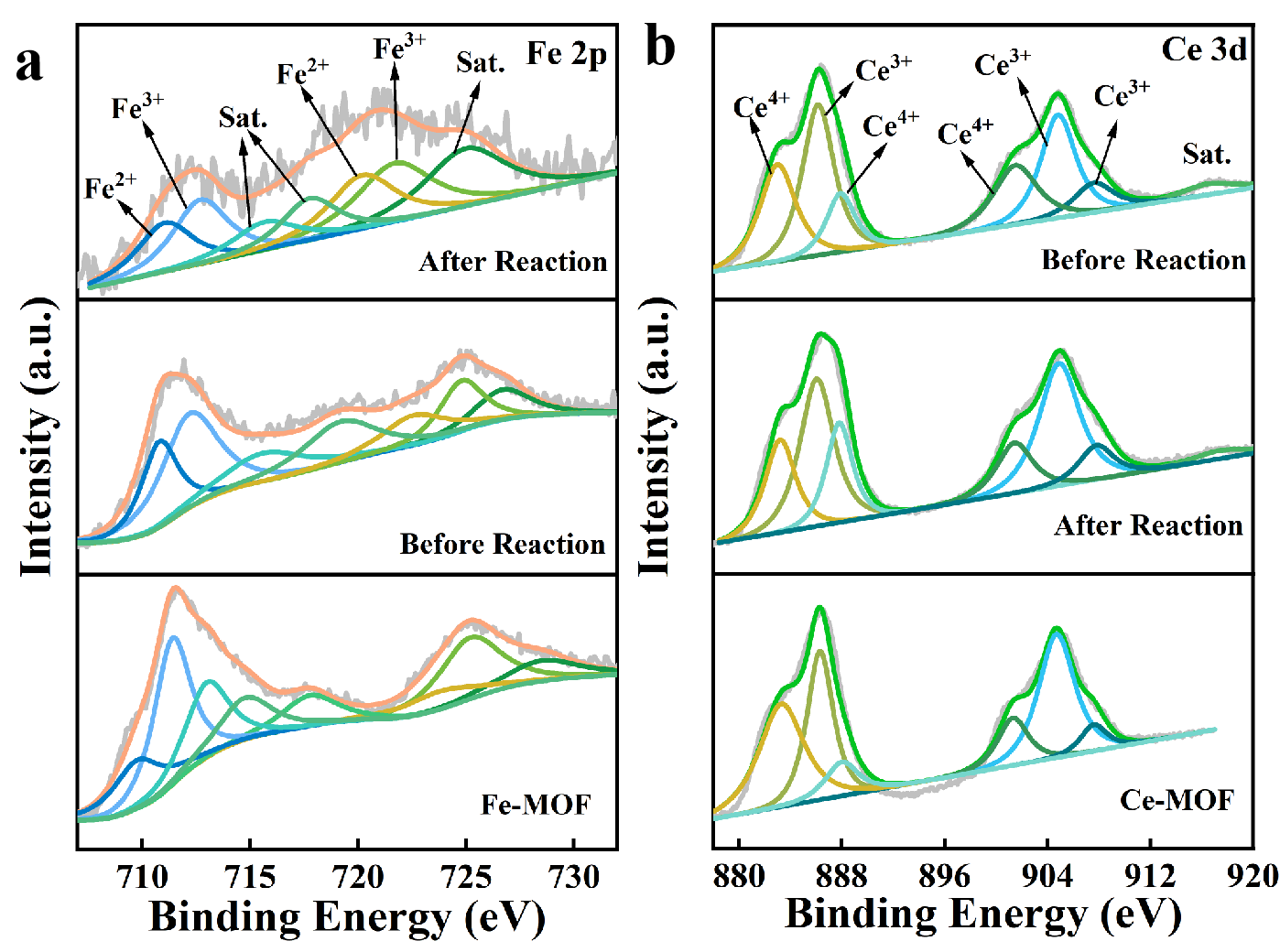


Fig. S9 XPS spectra of Fe 2p (a) and Ce 3d (b).


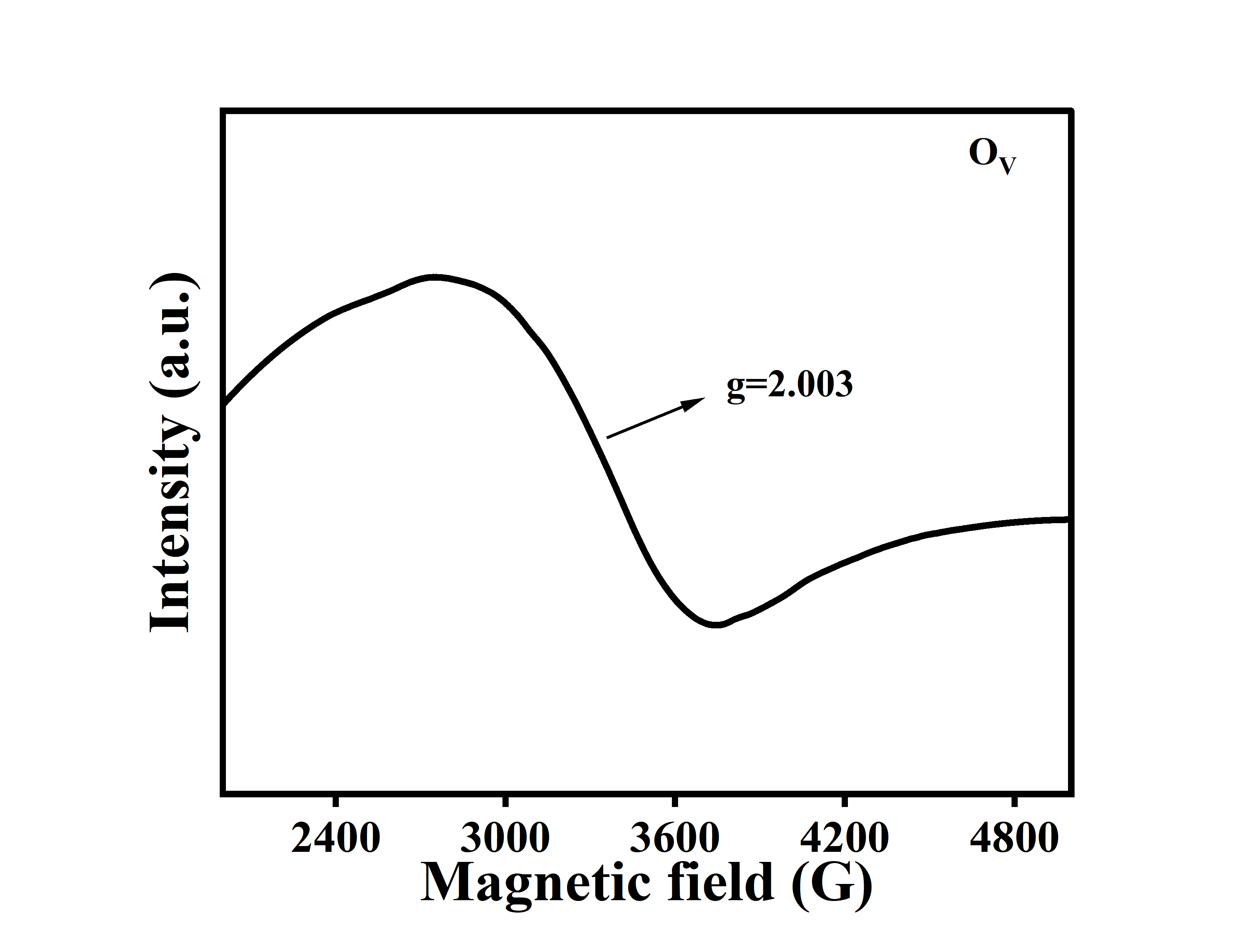


Fig. S10 EPR spectra of Ce/Fe-MOF.


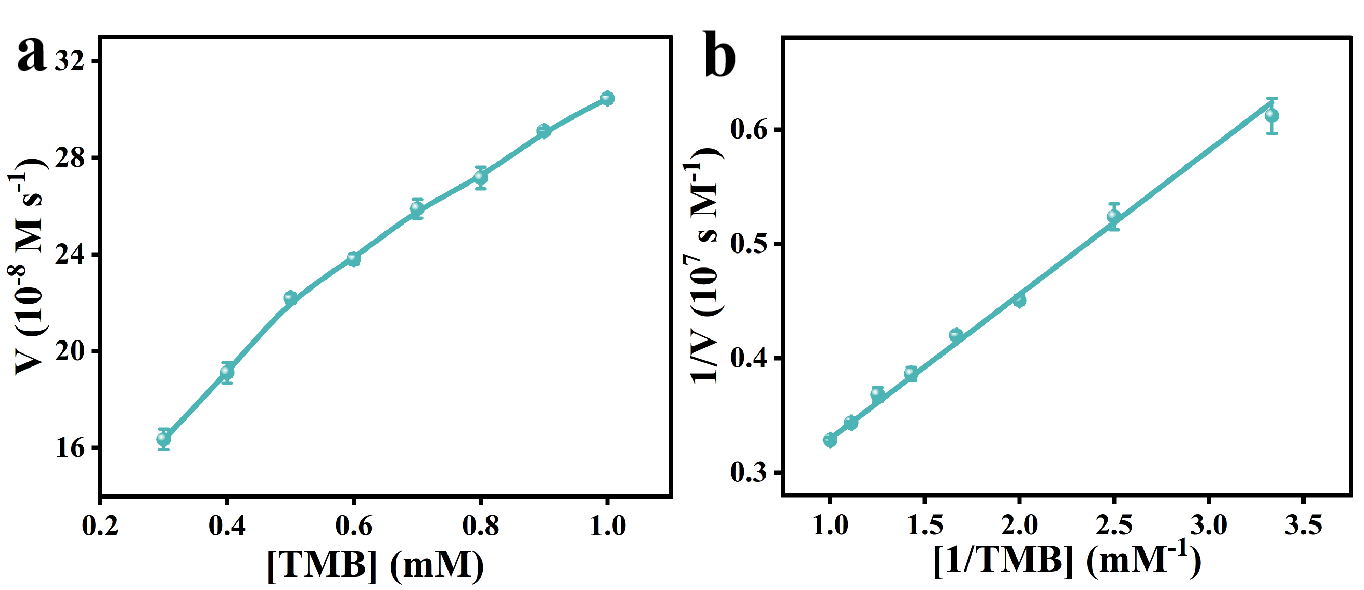
Fig. S11 Steady-state kinetic analysis of Ce/Fe-MOF (a). Double reciprocal plot of Ce/Fe-MOF (b).


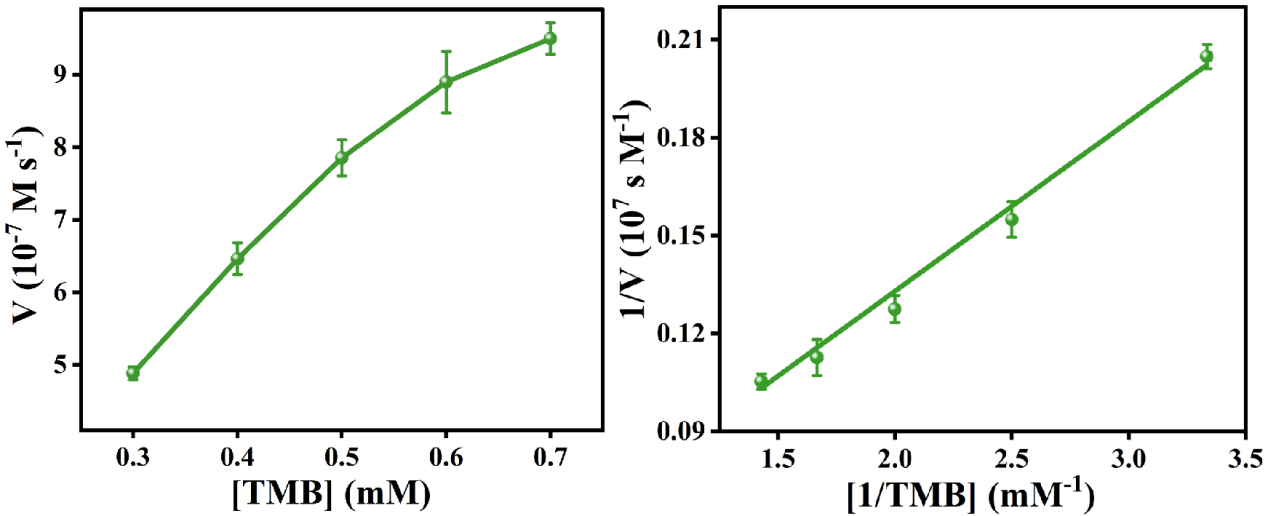


Fig. S12 Steady-state kinetic analysis of Fe-MOF (a). Double reciprocal plot of Fe-MOF (b).


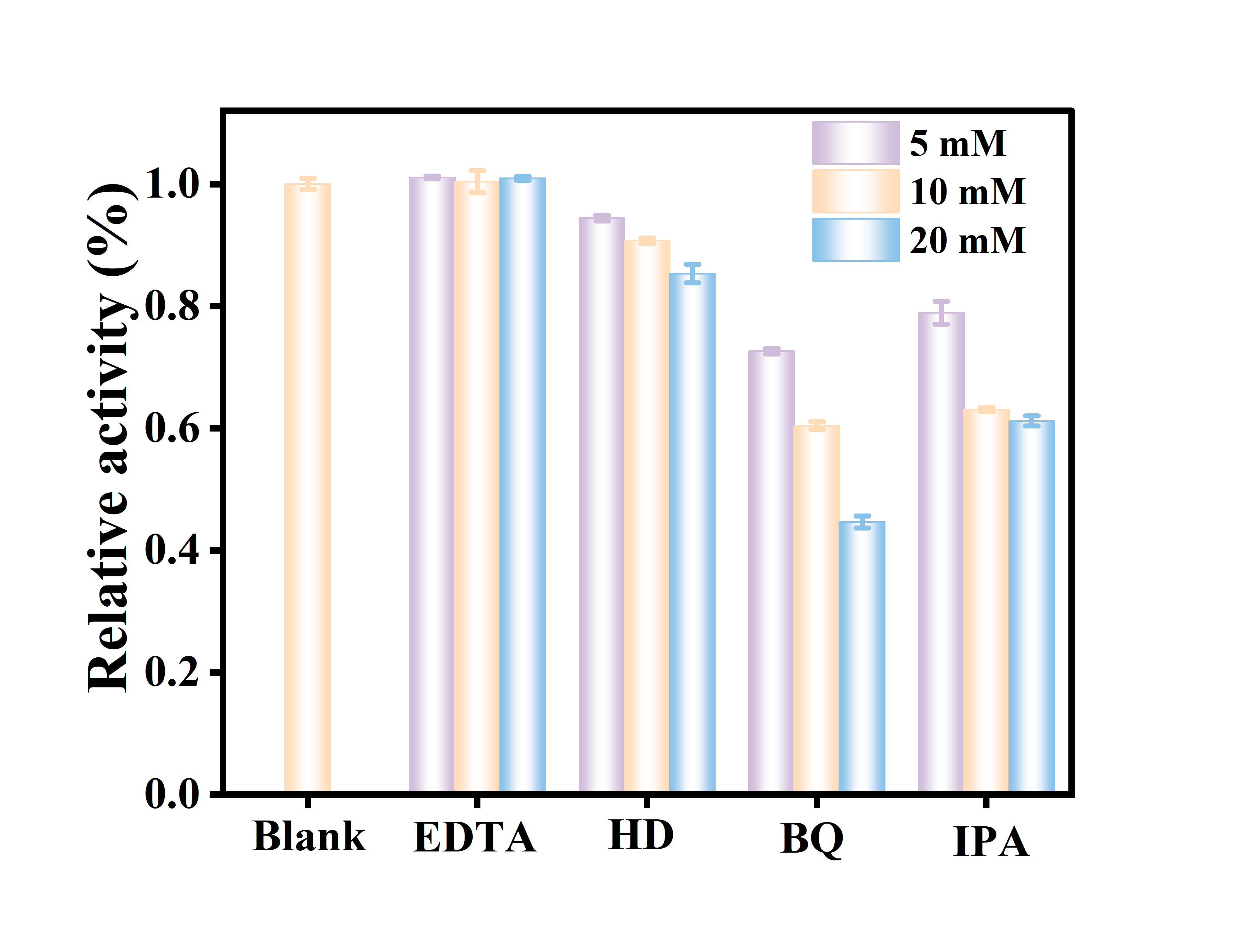


Fig. S13 Free radical capture of Ce/Fe-MOF.


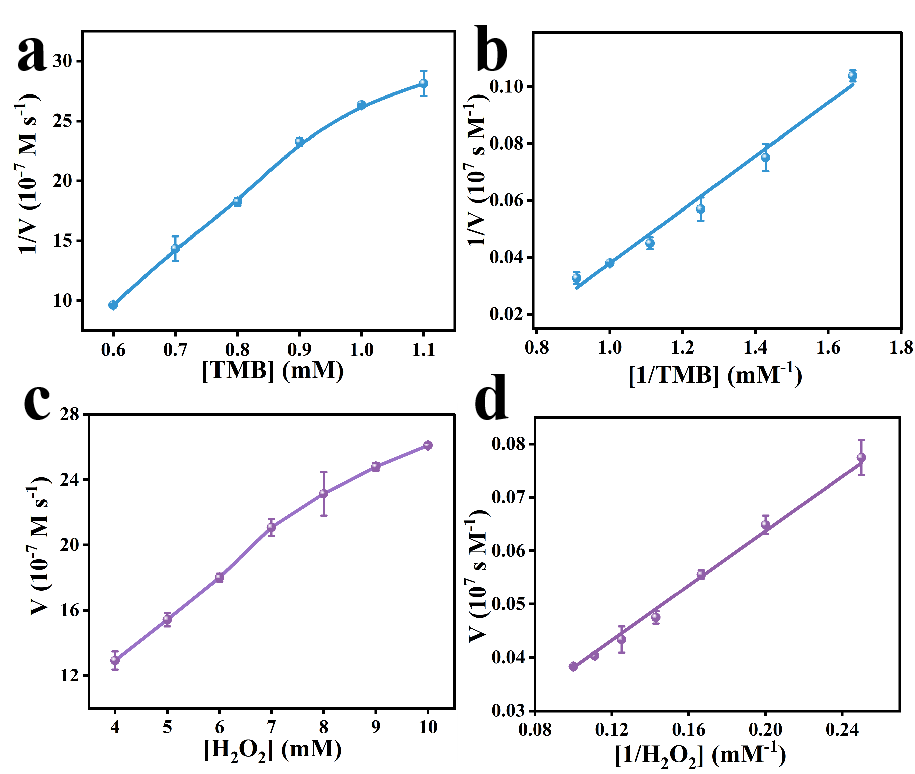


Fig. S14 Steady-state kinetic analysis of Ce/Fe-MOF. The velocity (v) changes of the reaction as the concentration of TMB (a) and H_2_O_2_ (c) changes. The double-reciprocal plot as the concentration of TMB (b) and H_2_O_2_ (d) changes.


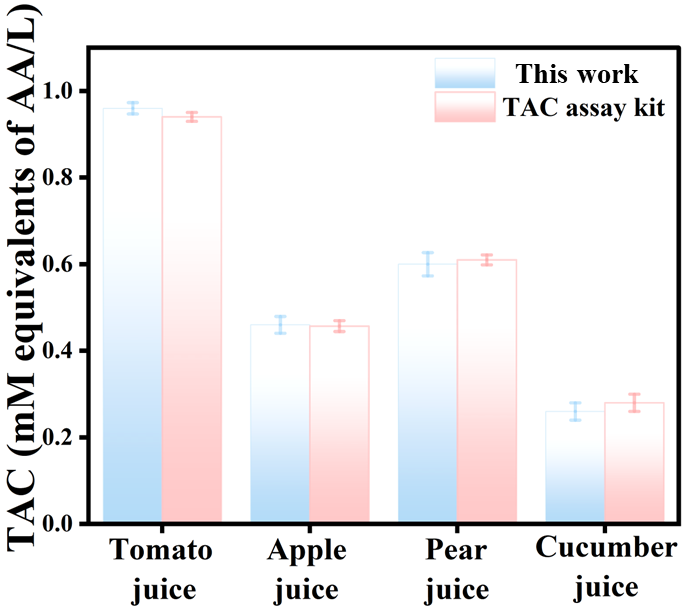


Fig. S15 TAC assessment by two methods in the corresponding samples.

**Section S-7: Supplementary Tables (S1-S4)**

Table S1 Comparison of various ratio of Ce^4+^/Ce^3+^ and Fe^3+^/Fe^2+^.

|  | Fresh Ce/Fe-MOF | Used Ce/Fe-MOF |
| --- | --- | --- |
| Ce^4+^/Ce^3+^ | 1.18 | 1.27 |
| Fe^3+^/Fe^2+^ | 1.49 | 0.94 |

Table S2 Comparison of various ratio of nanozymes.

| **Nanozymes** | ***K_m_* (TMB)/mM** | ***V_max_* (TMB)/10^-8^M·s^-1^** |
| --- | --- | --- |
| Ce/Fe-MOF | 0.62 | 49.1 |
| Fe-MOF | 1.79 | 34.5 |

Table S3 Comparison of various colorimetric methods for the determination of ascorbic acid.

| **Nanozymes** | **Analyte** | **Linear range (μM)** | **Detection limit (μM)** | **Ref.** |
| --- | --- | --- | --- | --- |
| MIL-88 | AA | 2.57-10.1 | 1.00 | (Gao et al., 2017) |
| Fe-MIL-88 | AA | 0.5-40 | 0.12 | (Chen et al., 2022) |
| MIL-53 (Fe) | AA | 28.6-190.5 | 15.00 | (Ai et al., 2013) |
| Pt/CeO_2_ | AA | 0.5-30 | 0.08 | (Liu et al., 2019) |
| RhB@MOF | AA | 1-25 | 0.31 | (Guo et al., 2019) |
| MOF (Co/2Fe) | AA | 8-80 | 8.00 | (Zhao et al., 2019) |
| Cu-MOF | AA | 10-50 | 1.40 | (Zhou et al., 2015) |
| Ce/Fe-MOF | AA | 2-60 | 1.30 | This work |

Table S4 Comparing the TAC of Ce/Fe-MOF colorimetric assay and Trolox/ORAC values for specific agricultural products.

| **Sample** | **TAC assay based on Ce/Fe-MOF (μM AA)** | **RSD (%)** | **ORAC**  **(μM TE)** | **RSD (%)** |
| --- | --- | --- | --- | --- |
| Tomato | 70.87 | 1.07 | 44.31 | 2.26 |
| Cucumber | 11.20 | 2.58 | 22.69 | 3.11 |
| Pear | 35.96 | 2.12 | 28.00 | 1.67 |
| Apple | 27.78 | 1.38 | 27.30 | 2.31 |

For the ORAC assay, by adopting the disparity between the area under the fluorescence decay curve in the presence of antioxidants and the area under the natural decay curve of fluorescence as a metric for antioxidant capacity, the results were quantified using the antioxidant substance Trolox as a standard.

Compared the TAC of Ce/Fe-MOF colorimetric assay and Trolox/ORAC values for four agricultural products, even though different methods depended on the different standard substrate used, the test results showed that tomato juice had the highest TAC value, then pear juice, apple juice, and cucumber juice the least.

**References**

Ai, L., Li, L., Zhang, C., Fu, J., & Jiang, J. (2013). MIL‐53 (Fe): a metal–organic framework with intrinsic peroxidase‐like catalytic activity for colorimetric biosensing. *Chemistry–A European Journal, 19*(45), 15105-15108. <https://doi.org/10.1002/chem.201303051>.

Chen, C.-x., Zhang, C.-h., Ni, P.-j., Jiang, Y.-y., Wang, B., & Lu, Y.-z. (2022). " Light-on" Colorimetric Assay for Ascorbic Acid Detection via Boosting the Peroxidase-like Activity of Fe-MIL-88. *Journal of Analysis and Testing*, 1-9. <https://doi.org/10.1007/s41664-021-00177-w>.

Gao, C., Zhu, H., Chen, J., & Qiu, H. (2017). Facile synthesis of enzyme functional metal-organic framework for colorimetric detecting H_2_O_2_ and ascorbic acid. *Chinese Chemical Letters, 28*(5), 1006-1012. <https://doi.org/10.1016/j.cclet.2017.02.011>.

Guo, L., Liu, Y., Kong, R., Chen, G., Liu, Z., Qu, F., Tan, W. (2019). A metal–organic framework as selectivity regulator for Fe^3+^ and ascorbic acid detection. *Analytical Chemistry, 91*(19), 12453-12460. <https://doi.org/10.1021/acs.analchem.9b03143>.

Liu, X., Wang, X., Qi, C., Han, Q., Xiao, W., Cai, S., Yang, R. (2019). Sensitive colorimetric detection of ascorbic acid using Pt/CeO_2_ nanocomposites as peroxidase mimics. *Applied Surface Science, 479*, 532-539. <https://doi.org/10.1016/j.apsusc.2019.02.135>.

Zhao, C., Xiong, C., Liu, X., Qiao, M., Li, Z., Yuan, T., Zhou, F. (2019). Unraveling the enzyme-like activity of heterogeneous single atom catalyst. *Chemical Communications, 55*(16), 2285-2288. <https://doi.org/10.1039/c9cc00199a>.

Zhou, E. L., Qin, C., Huang, P., Wang, X. L., Chen, W. C., Shao, K. Z., & Su, Z. M. (2015). A Stable Polyoxometalate‐Pillared Metal–Organic Framework for Proton‐Conducting and Colorimetric Biosensing. *Chemistry–A European Journal, 21*(33), 11894-11898. <https://doi.org/10.1002/chem.201501515>.
